# Projection of Gut Microbiome Pre- and Post-Bariatric Surgery To Predict Surgery Outcome

**DOI:** 10.1128/mSystems.01367-20

**Published:** 2021-06-08

**Authors:** Meirav Ben Izhak, Adi Eshel, Ruti Cohen, Liora Madar-Shapiro, Hamutal Meiri, Chaim Wachtel, Conrad Leung, Edward Messick, Narisra Jongkam, Eli Mavor, Shimon Sapozhnikov, Nitsan Maharshak, Subhi Abu-Abeid, Avishai Alis, Ilanit Mahler, Aviel Meoded, Shai Meron Eldar, Omry Koren, Yoram Louzoun

**Affiliations:** a The Mina and Everard Goodman Faculty of Life Sciences, Bar-Ilan University, Ramat-Gan, Israel; b Azrieli Faculty of Medicine, Bar Ilan University, Safed, Israel; c HY-Laboratories Ltd., Rehovot, Israel; d GENEWIZ, South Plainfield, New Jersey, USA; e Bariatric Surgery Unit, Surgery Division, Kaplan Medical Center, Rehovot, Israel; f IBD Unit and Bacteriotherapy Clinic, Department of Gastroenterology and Liver Diseases, Tel Aviv Medical Center and Sackler School of Medicine, Tel Aviv University, Tel Aviv, Israel; g Bariatric Surgery Unit, General Surgery Department, Tel-Aviv Medical Center and Sackler School of Medicine, Tel Aviv University, Tel Aviv, Israel; h Department of Internal Medicine C, Beilinson Hospital, Rabin Medical Center, Petach Tikva, Israel; i Bariatric Surgery, Asuta Medical Center, Ramat Ha'Chayal, Tel Aviv, Israel; j Bariatric Surgery Unit, The Baruch Padeh Medical Center, Poria, Israel; k Department of Mathematics, Bar-Ilan University, Ramat Gan, Israel; Mayo Clinic

**Keywords:** bariatric surgery, machine learning, obesity

## Abstract

Bariatric surgery is often the preferred method to resolve obesity and diabetes, with ∼800,000 cases worldwide yearly and high outcome variability. The ability to predict the long-term body mass index (BMI) change following surgery has important implications for individuals and the health care system in general. Given the tight connection between eating habits, sugar consumption, BMI, and the gut microbiome, we tested whether the microbiome before any treatment is associated with different treatment outcomes, as well as other intakes (high-density lipoproteins [HDL], triglycerides, etc.). A projection of the gut microbiome composition of obese (sampled before and after bariatric surgery) and lean patients into principal components was performed, and the relation between this projection and surgery outcome was studied. The projection revealed three different microbiome profiles belonging to lean, obese, and obese individuals who underwent bariatric surgery, with the postsurgery microbiome more different from the lean microbiome than the obese microbiome. The same projection allowed for a prediction of BMI loss following bariatric surgery, using only the presurgery microbiome. The microbial changes following surgery were an increase in the relative abundance of *Proteobacteria* and *Fusobacteria* and a decrease in *Firmicutes*. The gut microbiome can be decomposed into main components depicting the patient's development and predicting in advance the outcome. Those may be translated into the better clinical management of obese individuals planning to undergo metabolic surgery.

**IMPORTANCE** BMI and diabetes can affect the gut microbiome composition. Bariatric surgery has large variabilities in the outcome. The microbiome was previously shown to be a good predictor for multiple diseases. We analyzed here the gut microbiome before and after bariatric surgery and showed the following. (i) The microbiome before surgery can be used to predict surgery outcomes. (ii) The postsurgery microbiome drifts further away from the lean microbiome than the microbiome of the presurgery obese patients. These results can lead to a microbiome-based presurgery decision whether to perform surgery.

## INTRODUCTION

The human body is colonized by a wide variety of microorganisms, commonly referred to as the human microbiota. The gut microbiota is a complex ecosystem, which provides major functions to the host, such as regulation of metabolism, immune system modulation, and protection against pathogens (1, 2). The microbiome is strongly associated with host weight and sugar consumption, and as such, it serves as a proxy for nutrition and life habits and may also influence them. Such life habits may influence the total body mass and body mass index (BMI) in normal conditions, as well as after bariatric surgery.

Obesity and diabetes are world pandemics (3). Approximately 8 to 10% of the population develop complications of morbid obesity (BMI > 35), frequently coupled to some form of diabetes. According to the WHO, of the 57 million deaths in 2008 worldwide, 1.3 million were due to metabolic disorders, particularly those associated with obesity (3). Recently, the gut microbiome of obese individuals has been shown to differ from the microbiome of lean subjects (4). Experimental fecal transplants to mice demonstrated that transplantation of microbiome from obese individuals into lean mice turned them obese (5), showing the importance of the gut microbiome in regulating body weight. Opposite studies of turning obese mice into lean mice have not been successful, but one study demonstrated that certain bacteria can prevent weight gain in mice (6). Also, there have been reports of bacterial signatures which are correlated with metabolic disorders. For example, type 2 diabetes progression was characterized by a decrease in *Bifidobacteria* and *Verrucomicrobiae* levels. Moreover, Akkermansia muciniphila has been associated with reduced weight and insulin sensitivity improvement both in mouse models and humans (7).

The introduction of bariatric surgery as a method for losing weight has been rapidly adopted as the most efficient method for weight loss and for reducing blood sugar levels (8); however, it has drawbacks, including a range of possible complications from nutrition deficiencies to the occurrence of life-threatening conditions and an immense diversity in the weight loss rate and its maintenance (9). A few studies have shown microbiome changes after bariatric surgery. A recent systematic review (10) summarized the finding of 9 human studies and 12 animal studies and described an increase in the relative abundance of four major phyla: *Proteobacteria*, *Fusobacteria*, *Bacteroidetes,* and *Verrucomicrobia* as opposed to a decrease in the phylum *Firmicutes*. The dominant genera that changed were *Faecalibacterium*, *Lactobacillus*, and *Coprococcus*. An interesting finding was the increase in microbial diversity postsurgery (11). One of the mechanisms proposed for the effectiveness of bariatric surgeries is the changes in microbiome that influence the bile acids composition leading to metabolic improvement (12, 13). This can also occur the other way around, changes in bile acids, pH, and hormone levels lead to a change in the microbiome that affects energy homeostasis (14). However, the real potential of the microbiome as a tool not only for monitoring the procedure’s outcomes but rather predicting them in advance has not been explored. An attempt to study and test this potential can result in an essential tool that will assist in the decision whether to suggest to the patient to perform such surgery or to adopt a microbiome intervention approach as a part of the surgery preparative regimen.

## RESULTS AND DISCUSSION

**FIG 1:**
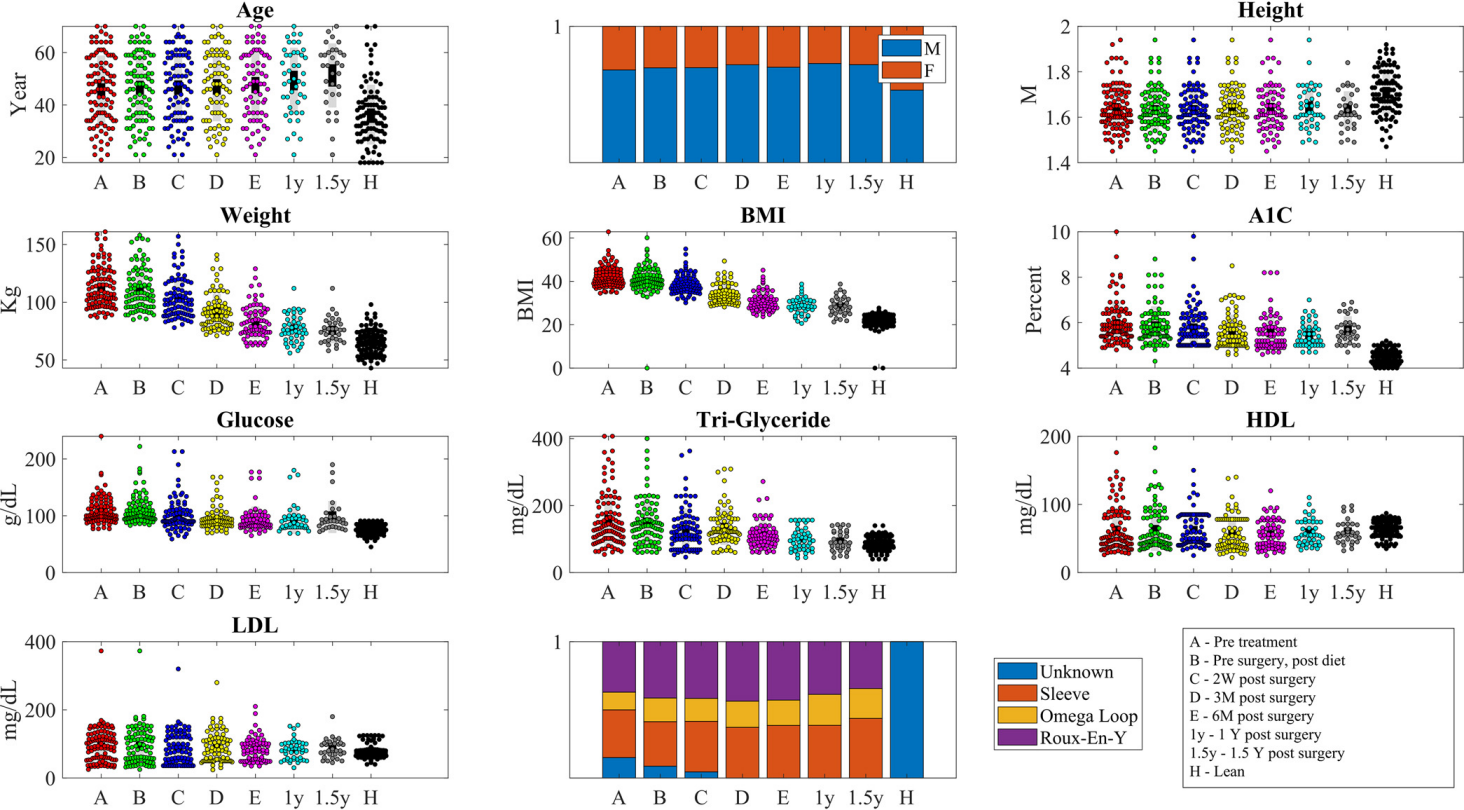
Distribution of age, gender, height, weight, BMI, A1C, glucose and triglyceride levels, and HDL and LDL levels at the samples taken in each group and time point (H is lean. All other samples are obese before [A and B] or after surgery [C, D, E, 1 year {1y}, 1.5y]. The time point codes are detailed in the bottom right part of the figure (W, week; M, month; Y, year).

To test the possibility of predicting bariatric surgery outcome, we analyzed 265 fecal samples from two main groups of patients: obese individuals who underwent bariatric surgery and naturally lean individuals. For the obese patients (BMI > 35), we sampled the microbiome at five time points (see Fig. S1 in the supplemental material)—one at enrollment (A, 66 samples), 3 weeks after a low carbohydrate diet, and immediately before the operation (B, 58 samples), and three time points following the surgery (2 weeks [C, 23 samples], 3 months [D, 22 samples], and 6 months [E, 9 samples]). Not all individuals were sampled at all time points. These were compared to 83 lean control individuals (BMI, 19 to 25) (for all details, see Materials and Methods). We collected BMI and sugar A1C information for the same patients in late time points up to a year and a half postsurgery to track their weight loss and the remission of diabetes. The obese individuals had Sleeve, Omega Loop, and Roux-en-Y surgeries, with an approximately equal fraction (Fig. 1).

10.1128/mSystems.01367-20.1 FIG S1 Experimental setup. The experiment tested the microbiome and different intakes (HDL, LDL, triglycerides, BMI, and A1C) of patients from two groups—obese individuals who underwent bariatric surgery and lean individuals. The lean individuals have been sampled once, while the obese patients were sampled in five time points—before the entire process (A), after low carbohydrate diet and before surgery (B), 2 to 3 weeks after surgery (C), 3 months after surgery (D), and 6 months after surgery (E). We further sampled the weight after 12 and 18 months. Download FIG S1, TIF file, 0.1 MB. Copyright © 2021 Ben Izhak et al. 2021 Ben Izhak et al. https://creativecommons.org/licenses/by/4.0/ This content is distributed under the terms of the Creative Commons Attribution 4.0 International license.

### Bariatric surgery significantly improved patients’ metabolic state.

The lean population was younger and had more females compared to the population who underwent surgery (36 ± 12 years versus 48 ± 12 and 50% males versus 29%). Overall, the patients’ mean BMI was reduced from 43.3 ± 6.8 (mean ± standard deviation [SD]) to 27.8 ± 1.5, which represents an average loss of 84.7% overweight (compared to BMI of 25), blood sugar levels were reduced from hemoglobin A1C of 6.5 ± 0.4 to 5.8 ± 0.75 or from blood sugar levels of 125 ± 11 g/dl to 95.4 ± 10, triglyceride levels decreased from 183 ± 20 mg/dl to 102 ± 13. All parameters described are significantly (*P* < 0.001) lower from their starting point and not different from those of the lean controls (Fig. 1).

### 16S data processing steps.

**FIG 2:**
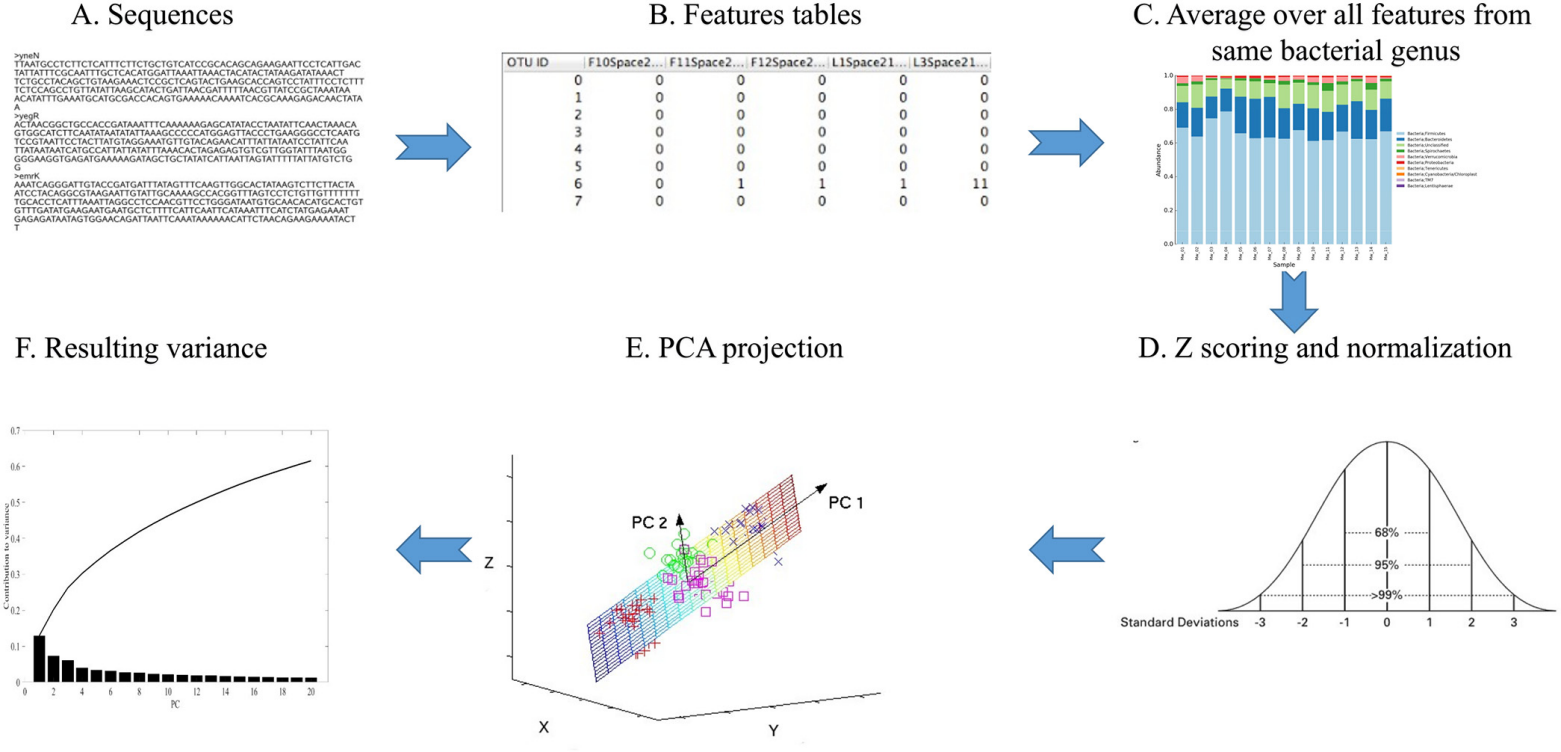
Outline of analysis (from top left to top right and then from bottom right to bottom left). First, the Fastq sequences are quality controlled. The good quality sequences are translated to features using QIIME2. To homogenize the description level, the feature levels belonging to the same genus in a given sample are averaged to the genus level. (Bottom) The sample distribution is heavy-tailed. It is thus log transformed with a minimal value (0.1) added to each feature level to avoid log of zero values. The results are then z scored by removing the average and dividing by the standard deviation of each sample. The dimension of the z-scores is further reduced using PCA. The first eight PCs explain approximately 50% of the total variance (bottom left panel).

The gut microbiome of all donors was analyzed using 16S rRNA gene sequences (emphasizing the 16SMetaVx.V2) (15), and feature tables were produced using QIIME2 (16). If a feature was present in only some of the samples, it was given a value of 0 in all other samples. The feature tables were then merged to the genus level. Taxa appearing in less than five samples (each sample is from one time point from one host) were removed. The resulting values had a scale-free distribution, which often masks large changes in relative frequencies of rare bacteria. To handle that, we log transformed all feature values and added a small constant value (0.1) to avoid a log of zero values. This allows for a narrower distribution of values (Fig. 2D). The results were then z-scored. Finally, given the very high correlation between the relative abundance of different bacteria (Fig. 2E), we projected the z-scored bacterial expression levels at all time points and in the lean population to principal components, which capture most of the variance in the bacterial diversity (Fig. 2F).

The aim of the first step was to homogenize the description level and reduce the dimension. Since multiple features are associated with the same bacteria and some features are associated with different levels of classification, we averaged all features associated with the same species in each donor (Fig. 2A to C). It is important to note that while information is lost in the process, such a process is essential for the following machine learning. We have previously demonstrated, in multiple microbiome-based machine-learning studies (17–21), that averaging over all features representing the same genus or species improved the prediction accuracy.

### Microbiome-based classification allows distinction between groups.

**FIG 3:**
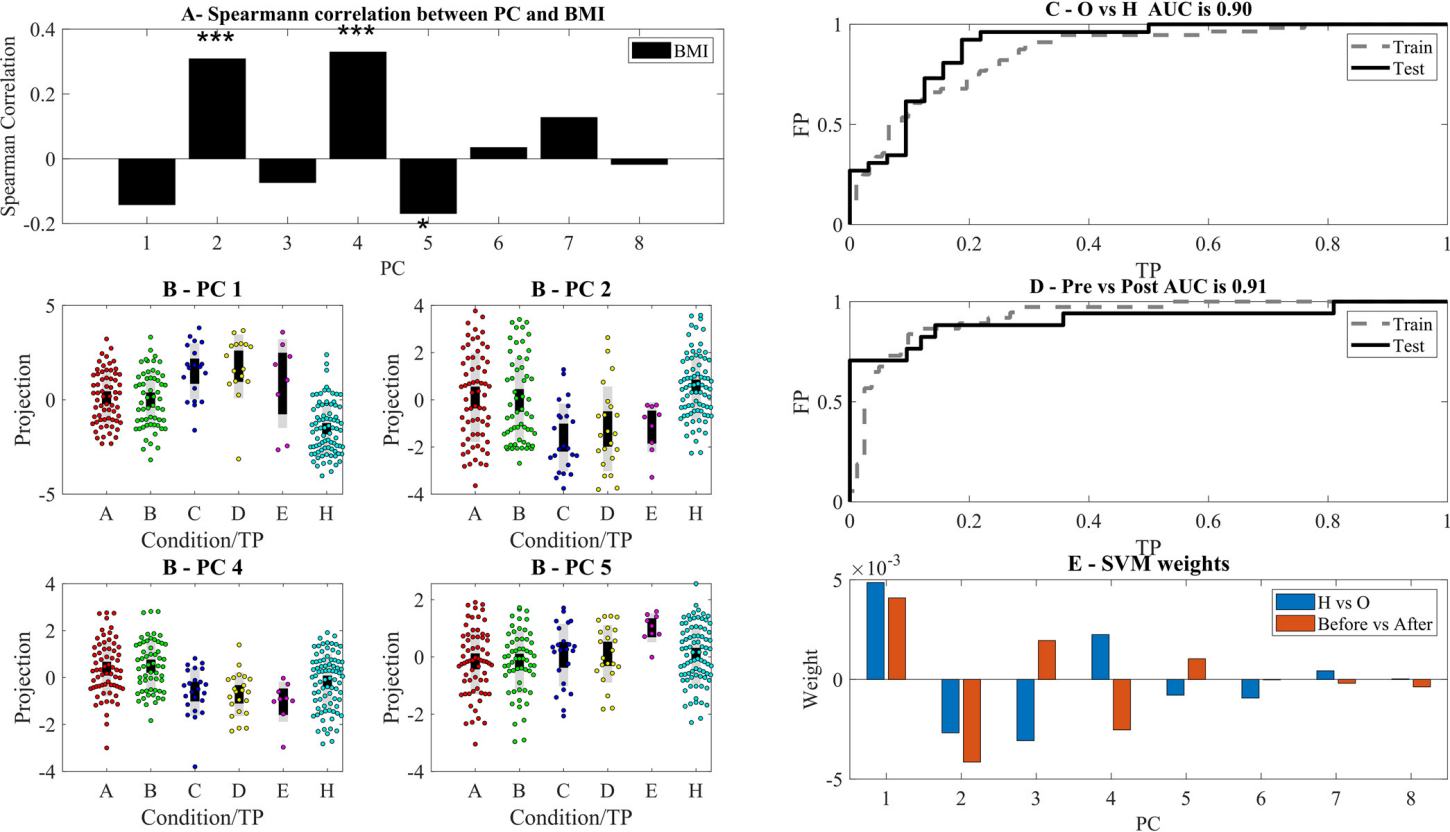
(A) Spearman correlation between BMI of samples and the eight highest variance PCs. ***, **, and * represent significance levels of 0.001, 0.01, and 0.05, respectively (in this and all following figures). PC2, PC4, and PC5 are the most correlated with BMI. (B) Projection of the significant PCs on the different stages. One can see in all PCs a clear difference between the H and obese states. Following surgery, the projection is farther away from the H state than before. Note that we do not have microbiome samples from the latest time points. TP, time point. (C to E) Receiver operating characteristic (ROC) curve of linear SVM classification using the projections on the first 8 PCs of the H versus O and within the O group before versus after surgery (C and D) and the resulting weights (E).

The projection on the first principal vectors (principal component 1 [PC1], PC2, PC4, and PC5) delineates axes separating the obese individuals from the lean individuals (Fig. 3A and B). The clear separation of the projections on the first PCs agrees with observed major differences in the microbiome of lean and obese individuals. The large BMI difference between groups (BMI > 35 in obese versus BMI of <25 in lean) is translated to a large difference in the microbiome.

However, the cohort was composed of two populations of lean individuals: the untreated lean, and the lean as a result of surgery or diet. We tested whether diet or bariatric surgery pushes the microbiome back toward the lean profile. The results were surprisingly opposite (Fig. 3B). The distance between the projection on the first PC of the postdiet and postsurgery and the lean profile kept increasing and reached a maximum after a year.

**FIG 4:**
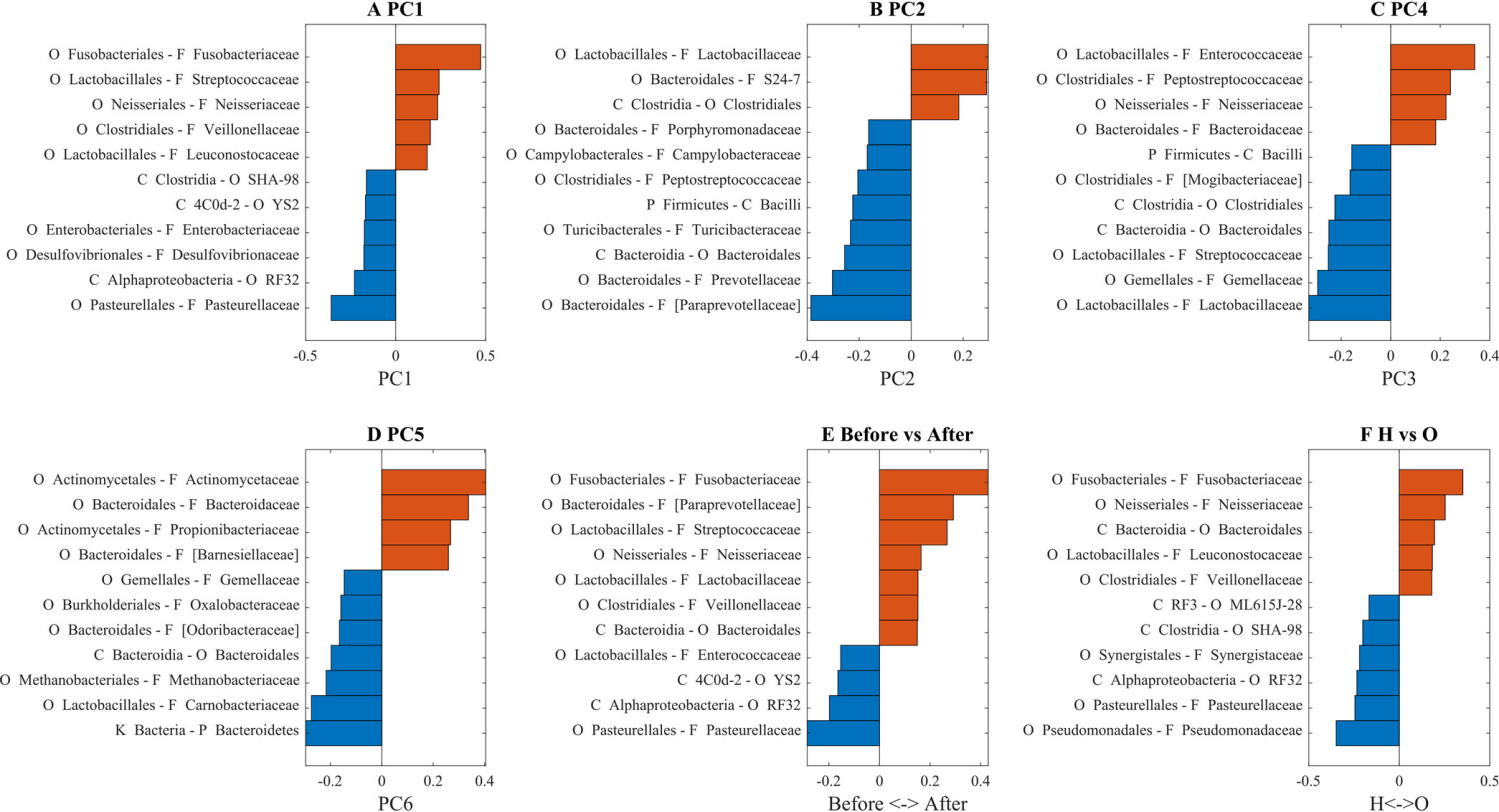
(A to D) Bacterial composition of PC1, PC2, PC4, and PC5. (E and F) Weights of linear SVM classifier for H versus O and before versus after surgery. Only the top 15% of features are presented (based on their absolute weights).

We projected back the correlations between the PC and the state/BMI to the original features and found bacteria that are correlated with BMI (PC1, PC2, PC4, and PC5; Fig. 4A to D, respectively), the features that change significantly after surgery compared to before surgery (Fig. 4E) and the features that are over- and underrepresented in obese individuals compared to healthy individuals (Fig. 4F).

The large difference between these projections allowed for a simple classification even with a linear support vector machine (SVM) of lean versus obese and pre- versus postsurgery samples (Fig. 3C and D). The main contributions to both classifiers were from PC1, PC1 to PC5 for the healthy (H) versus obese (O) (Fig. 3E for contribution and Fig. 4A to D for composition). Note that higher test area under curve (AUC) could be obtained by nonlinear classifiers. However, the linear classifier gave a clear picture of the contribution of each PC to the microbiome development.

**FIG 5:**
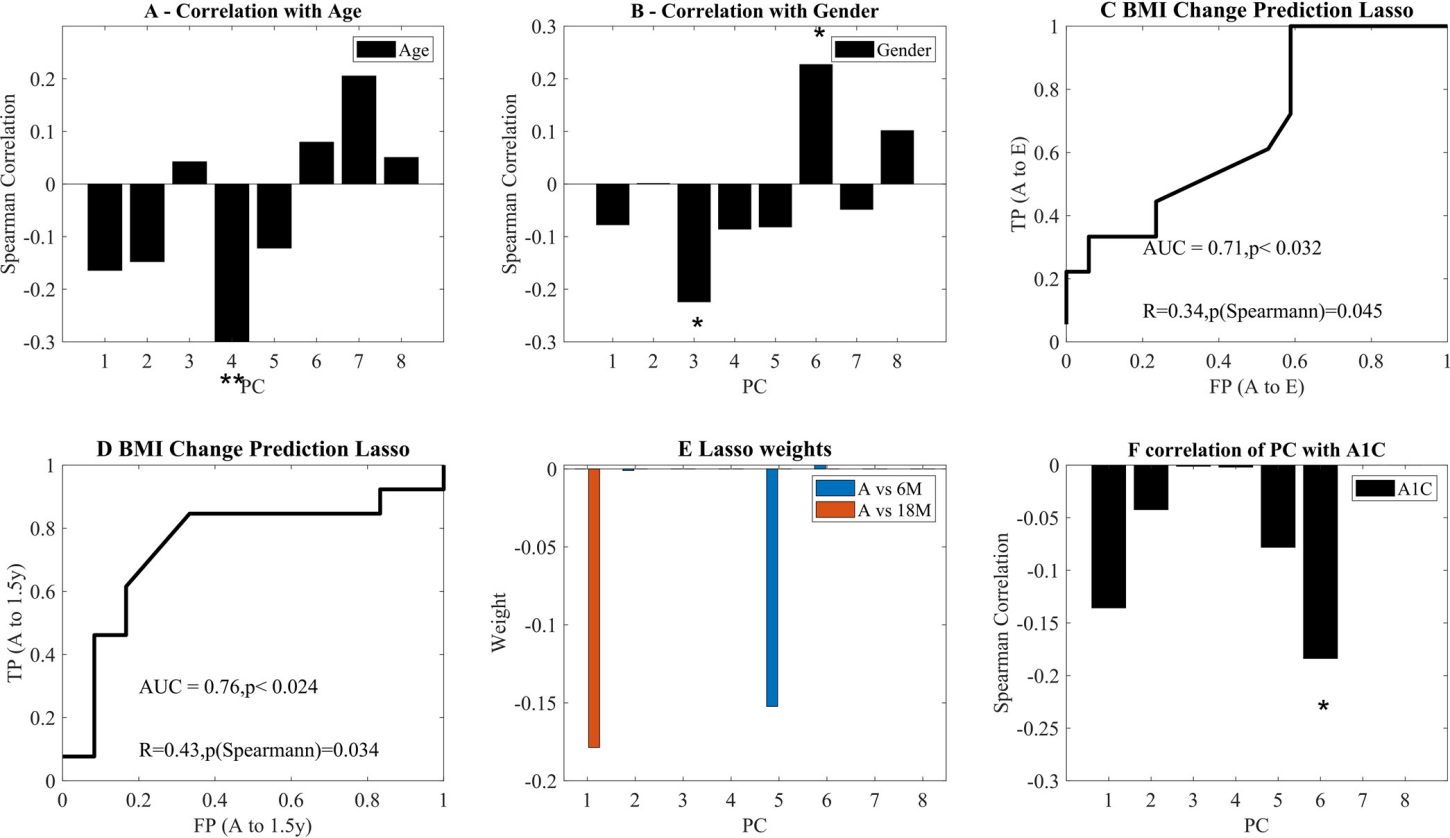
(A and B) Correlation of age, gender (represented as 0 for male and 1 for female) and BMI with the PC. (C and D) ROC curves of future BMI change based only on the PC at point A. The binary predicted change is above or below the median change. The ROC curves for above and below median change in BMI, using a LASSO regression and a LOO validation. C is for 6 months and D is for 18 months postsurgery. The first *P* value is the ROC curve *P* value compared with AUC of 1,000 scrambling of the predicted values. The second P is the Spearman correlation *P* value between predicted and actual change. (E) Average regression weights over all LOO learning sessions. The only nonzero coefficients are the fifth PC for 18M and the first PC for the 6M. (B to F) Average weights of coefficients in the LASSO regression for plots C and D over all LOO predictions. In all plots, * and ** represent significance levels of *P* < 0.05 and *P* < 0.01, respectively.

To test for possible confounding effects, we assessed whether the observed changes in the profile may result from age or gender or whether they are related to the total BMI. There was a limited correlation with age, and no significant correlations with gender and age of all other PCs (Fig. 5A and B).

### Surgery outcome prediction model.

We next tested whether the same decomposition can be used to predict a future change in BMI. We performed an L1 (Lasso) regression of the projection on the first PCs of the A point and the change in BMI between point A and 6/18 months after surgery. The prediction was performed using only presurgery and prediet (point A) samples and tested using a leave-one-out (LOO) methodology, and the Spearman correlations on the test values between the predicted change and the observed change (on the LOO test), as well as the area under curve of a predictor of whether a patient will have a more/less than average reduction in BMI. The AUC and correlations are significant for both points (Fig. 5C and D). The AUC and correlation for 12 months (12M) are also above random but do not reach significance (data not shown). When looking at which features are contributing to weight loss at 6 months, PC1 and PC5 were the only contributors (Fig. 5E). Note that the prediction was based on the microbiome presurgery, so that these results could not be an effect of surgery type.

Another possible candidate to affect the microbiome is the sugar level. We tested the correlation between the projections on the principal component analysis (PCA) and the A1C. Indeed, a negative correlation is found between A1C and PC6 (Fig. 5F). This correlation might be used for predicting diabetes in healthy subjects both from risk groups and in general.

### Postsurgery microbiome’s dynamics.

To better understand the microbiome’s dynamics following surgery, we tracked the development of the main phyla, following diet and surgery. The main observed changes are the increase in *Proteobacteria* and *Fusobacteria* frequency and the decrease in *Firmicutes* (Fig. S2). Within *Proteobacteria*, *Beta*- and *Gammaproteobacteria* were the main elevated classes, while in *Firmicutes*, *Bacilli* increased over time following surgery (Fig. S3).

10.1128/mSystems.01367-20.2 FIG S2 Normalized log expression level as a function of time point merged at the phylum level. Download FIG S2, TIF file, 0.3 MB. Copyright © 2021 Ben Izhak et al. 2021 Ben Izhak et al. https://creativecommons.org/licenses/by/4.0/ This content is distributed under the terms of the Creative Commons Attribution 4.0 International license.

10.1128/mSystems.01367-20.3 FIG S3 Normalized log expression level as a function of time point merged at the class level. Download FIG S3, TIF file, 0.4 MB. Copyright © 2021 Ben Izhak et al. 2021 Ben Izhak et al. https://creativecommons.org/licenses/by/4.0/ This content is distributed under the terms of the Creative Commons Attribution 4.0 International license.

We next tested which bacteria are associated with changes in other bacteria. To compute that, we performed a correlation between the change in each bacterial log frequency versus the other bacterium’s value. For example, given two time points, compute the correlation between the log frequency of bacterium *x* at time point 1 and the change in log frequency between time points 1 and 2 of bacterium *y*. We define a feature correlated with a positive/negative change in another feature to have a positive/negative interaction with this feature, leading to an interaction network (Fig. S4). For example, the presence of members of the family *Pasteurellaceae* at a given time point has a negative influence on the presence of members of the families *Ruminococcaceae*, *Xanthomonadaceae*, *Pasteurellaceae* and members of the *Bacilli* class at a later time point. Such a network demonstrates how dynamic the microbiome is from one point to the next and how the presence of certain taxa influences their increase/decrease in abundance in the next time point. Further research is needed to tease out the biological mechanisms behind these multiple interactions.

10.1128/mSystems.01367-20.4 FIG S4 Interaction map computed from changes in the dynamics of the log normalized feature expression level. Red arrows represent negative interactions. Gray arrows represent positive interactions. Download FIG S4, TIF file, 0.2 MB. Copyright © 2021 Ben Izhak et al. 2021 Ben Izhak et al. https://creativecommons.org/licenses/by/4.0/ This content is distributed under the terms of the Creative Commons Attribution 4.0 International license.

### Conclusions.

To summarize, we have shown that the decomposition of the relative bacterial frequency (as represented by log value) represents different aspects of the donors. This decomposition highlights that people who lost weight after bariatric surgery have a very different microbiome composition compared to people who are “naturally” lean. Furthermore, the more weight they lose, the more their microbiome profile differs not only from their starting profile as obese but also from naturally lean people.

Some of the effects of bariatric surgery are changes in bile acid metabolism and gastric pH (14). These changes may in turn influence the microbiota. When analyzing the microbial dynamics pre- and postsurgery, we observed a dramatic increase in the *Proteobacteria* and *Fusobacteria* phyla which fit previous observations (22–33). *Proteobacteria* are known to increase in disease states and to have proinflammatory characteristics (34). However, in the case of bariatric surgery, Tremaroli et al. and others have attributed this increase to changes in the pH that follow the surgery in humans, mice, and rats (22, 26, 35–38). The increase in *Fusobacteria* resulted from an increase in the relative abundance of *Fusobacterium*. Recently, Ilhan et al. reported that an increase in levels of *Fusobacterium* following surgery was negatively correlated with levels of primary bile acids such as glycocholic acid, taurodeoxycholic acid, glycochenodeoxycholic acid, and taurochenodeoxycholic acid and secondary bile acids such as glycodeoxycholic acid, taurolithocholic acid, glycolithocholic acid, hyodeoxycholic acid, taurodeoxycholic acid, and lithocholic acid (39). As in our study, the increase in *Proteobacteria* and *Fusobacteria* persisted 1 year postsurgery as reported by Palleja et al. (27).

Moreover, one can predict in advance whether surgery will succeed in reducing BMI and whether a subject has diabetes using only presurgery samples. The PCs are determined by the composition of the studied populations, and the analysis of different populations may highlight different possible projections of the microbiome composition. Given the large fraction of people regaining weight postsurgery, such a tool to aid decisions can be crucial. Note that the current prediction is purely based on the microbiome, as it is the main focus of this analysis. For clinical applications, other meta-information could be used to improve the decision.

## MATERIALS AND METHODS

### Patients and regulation.

Patients were enrolled in the obesity control/bariatric surgery clinics of four medical centers in Israel – Kaplan Medical Center (KMC), Rabin Medical Center (RMC), Tel Aviv Medical Center (TMC), and Poria Medical Center (PMC) during December 2015 to November 2018. The ethics committees of each of the respective medical centers approved the study and its amendments, and each patient and control signed written informed consent. Inclusion criteria were: ages 18 to 70, no antibiotic treatment in the 2 months before enrollment, no previous bariatric or major gut/stomach operation. One-third of the patients had diabetes (type 1 and type 2) and were treated with either insulin (type 1) or other drugs (metformin or others). Patients with ulcers who were treated by proton pump inhibitors (PPI) were not enrolled.

Naturally lean control individuals were defined as having a BMI of 19 to 25 and are healthy controls recruited from the same population. The controls had no diabetes (hemoglobin A1C < 5.0), BMI of 19 to 25, and had no major medical and endocrine complications. Obese individuals had BMI values of 35 and above, with and without active diabetes type 1 or 2 that is treated with medications. A total of 83 lean control and 66 obese individuals were tested at multiple time points.

Naturally lean control individuals gave one fecal, blood, and urine sample. Obese/diabetic people gave five samples of each at the following time points: time of enrollment (group A), 3 weeks after a low carbohydrate diet and immediately before the operation (group B), 2 weeks (group C), 3 and 6 months after the operation (groups D and E, respectively). In all visits, the patients' weight, blood, and urine test results, medications, and general health issues were noted. The obese group also provided weight values and blood test results at 1 and 1.5 years after the operation. At the time of analysis, not all patients had completed their course of testing and evaluation.

Blood tests included the standard complete blood count (CBC) test list and the blood biochemistry test following 12-h fasting (of relevance here triglycerides, low-density lipoproteins [LDL], high-density lipoproteins [HDL], blood glucose). Also, patients provide samples for the standard hemoglobin A1C (HbA1C) test.

### DNA extraction.

Fecal samples were stored in Flora prep tubes (Admera Health, NJ, USA) with a proprietary bacterial DNA preservation medium, allowing for storage at room temperature. The samples were brought into the next generation sequencing (NGS) lab within 1 to 2 days after collection and were subjected to DNA extraction, purification, and cleaning. DNA was extracted from the stool sample using the PowerSoil DNA extraction kit (MoBio, Carlsbad, CA) according to the manufacturer’s instructions. Purified DNA was used for PCR amplification of the variable V3 and V4 regions (using MetaVx.2 system developed with GENEWIZ (Plainfield, NJ) of the 16S rRNA gene as was previously described in U.S. patent 9,745611 and by Caporaso et al. (40). Amplicons were purified using AMPure magnetic beads (Beckman Coulter, Brea, CA) and subsequently quantified using Qubit double-stranded DNA (dsDNA) quantification kit (Invitrogen, Carlsbad, CA). Equimolar amounts of DNA from individual samples were pooled and sequenced using the Illumina MiSeq platform and V2 500 cycle kit.

### DNA amplification.

Briefly, the amplification method includes using multiple sets of overlapping primers and the generation of multiple frame-shifted amplicons, which increase taxonomically diverse sequence amplification. The primers used in the present work are listed in Table 1 and are designed to amplify the variable regions V3 and V4 of the bacterial 16S rRNA. As can be seen from Table 1 of U.S. patent 9,745,611, these primers are capable of amplifying sequences from bacterial gut microbiome to identify rare species which may be difficult to amplify by similar kits based on 16S rRNA V4 region sequence amplification. The use of this procedure enabled the identification of bacterial taxonomic entities below 0.2% of the population and the detection of rare species.

**TABLE 1:** Primer sequences

Primer	Sequence^*a*^	SEQ ID no.^*b*^
U341F-p5	ACACTCTTTCCCTACACGACGCTCTTCCGATC TNCCTACGGGRSGCAGCA	1
E343F-p5	ACACTCTTTCCCTACACGACGCTCTTCCGATC TNTACGGRAGGCAGCAG	2
E347F-p5	ACACTCTTTCCCTACACGACGCTCTTCCGATC TNGGAGGCAGCAGTRRGGAAT	3
E347F-p5-n	ACACTCTTTCCCTACACGACGCTCTTCCGATC TNNGGAGGCAGCAGTRRGGAAT	4
A349F-p5	ACACTCTTTCCCTACACGACGCTCTTCCGATC TNGYGCASCAGKCGMGAA	5
E802R-p7	GACTGGAGTTCAGACGTGTGCTCTTCCGATC TNTACNVGGGTATCTAATCC	6
E803R-p7	GACTGGAGTTCAGACGTGTGCTCTTCCGATC TNCTACCRGGGTATCTAATCC	7
P803R-p7	GACTGGAGTTCAGACGTGTGCTCTTCCGATC TNCTACCRGGGTATCTAAGCC	8
E806R-p7	GACTGGAGTTCAGACGTGTGCTCTTCCGATC TNGGACTACHVGGGTWTCTAAT	9
A806R-p7	GACTGGAGTTCAGACGTGTGCTCTTCCGATC TNGGACTACVSGGGTATCTAAT	10
U805R-p7	GACTGGAGTTCAGACGTGTGCTCTTCCGATC TNGACTACHVGGGTATCTAATCC	11
U805R-p7-n	GACTGGAGTTCAGACGTGTGCTCTTCCGATC TNNGACTACHVGGGTATCTAATCC	12

a According to IUPAC nucleotide code: K is G/T, M is A/C, R is A/G, Y is C/T, S is C/G, W is A/T, V is A/C/G, H is A/C/T, and N is A/G/C/T.

b Sequence identification number.

### Microbiome analysis.

Microbial communities were analyzed using QIIME2 (16). Single-end sequences were demultiplexed by per-sample barcodes and error corrected by Divisive Amplicon Denoising Algorithm (DADA2) (41), primers were trimmed off, and single-end reads were truncated to ≥160 bp. Feature sequences were aligned against Greengenes database v13_8 (42) with a similarity of 99% or greater for taxonomic annotation. Finally, the following contaminants have been removed from the feature table: Thermi, S24-7, and Chloroplast (43). The feature tables represent the frequency of each representative feature in each sample.

### Normalization.

Features were merged to the genus level by averaging over all features assigned to the same genus. Given the large variation in feature values, we transformed these values to z scores by adding a small value to each feature level (0.1) and calculating the 10-basis log of each value. Statistical Whitening was then performed on the table, by removing the average and dividing by the standard deviation of each feature.

### Machine learning.

Supervised learning was performed on the normalized and merged version of the 16S rRNA feature table to recognize patterns in the data. Principal component analysis (PCA) was performed using Python version 3.5 and its package sklearn. A two-tailed *P* value of less than 0.05 was considered to indicate statistical significance. A LASSO regression was performed over the projection of the normalized features on the principal components (PCs) of the PCA to predict future BMI change. Leave-one-out cross-validation method was performed. More complex methods were not used to limit overfitting, given the limited number of samples.

### Statistical analysis.

All correlations studied here are Spearman correlations. *P* values of ROC curves are computed using scrambling the classes (positives or negative) of the samples and computing the AUC of 1,000 scrambles. The real AUC was compared to the 1,000 scrambles. Benjamini Hochberg correction was performed when multiple correlations were computed for each PC (for example when correlating age with PCs of microbiome projection).

### Microbiome dynamics.

To understand the microbiome dynamics, we used two analysis methods. In the stand-alone method, we merged features to the phylum level and then tracked the changes within each phylum and within each class in each phylum. We then detected class with a consistent trend in time.

In the network analysis, we computed the regression of each relative change in each log expression level of each bacterium over the value of all other bacteria. Formally, given log-normalized bacterial frequencies $\left(x_{i,t}\right)$ for bacterium *i* in time point *t*, we defined $y_{i,t}=x_{i,t+1}-x_{i,t}$ and performed a regression $y_{i,t}\approx \sum^{\;}\beta_{ji}x_{j,t}.$ We used only the coefficients that were significant at the *P* = 0.05 level after a Bonferroni multiple measurement correction. We then produced a network out of all significant coefficients, where β*_ji_* is represented by an edge between *i* and *j*. There was one main (weak) connectivity component. We then presented as a network all bacteria belonging to this main component.

### Data availability.

16S data have been deposited at EBI with accession number ERP122895.
