# Supplementary figures and images for: Projection of Gut Microbiome Pre- and Post-Bariatric Surgery To Predict Surgery Outcome
Source: mSystems. 2021 Jun 8;6(3):e01367-20. doi: 10.1128/mSystems.01367-20 (PMC8269264; doi:10.1128/mSystems.01367-20)

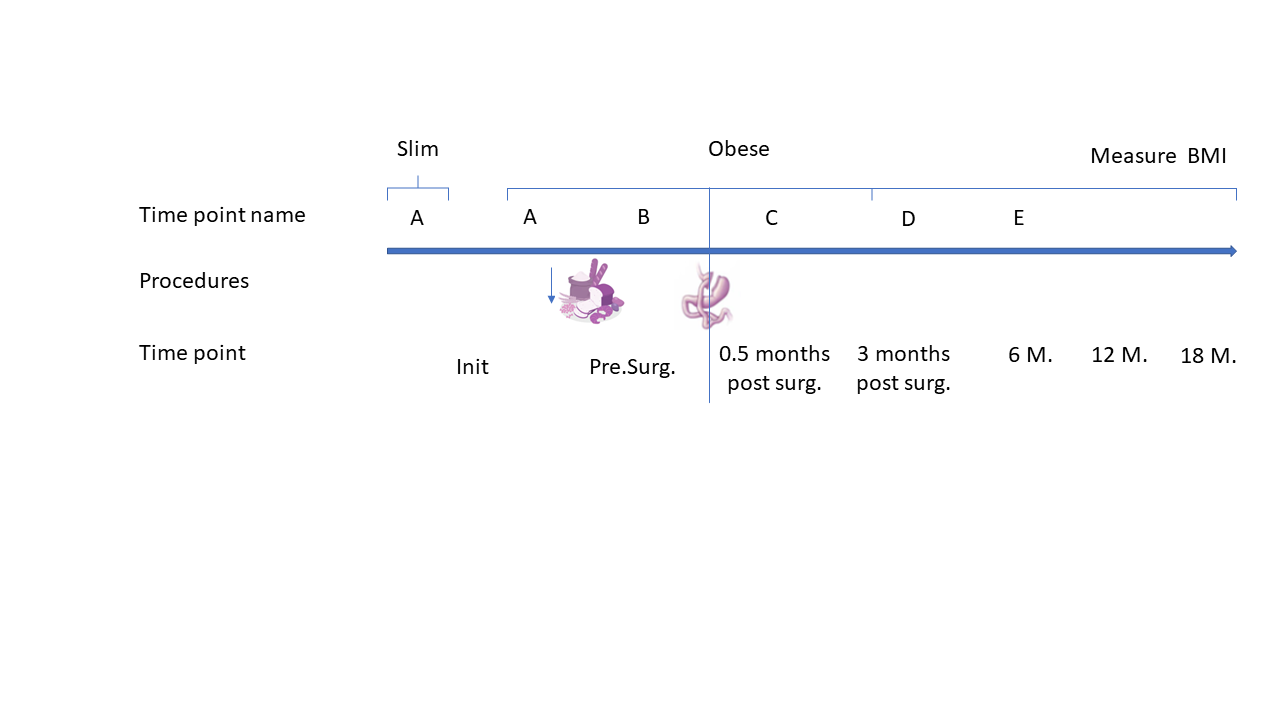

Supplement: FIG S1 [file msystems.01367-20-sf001.tif]

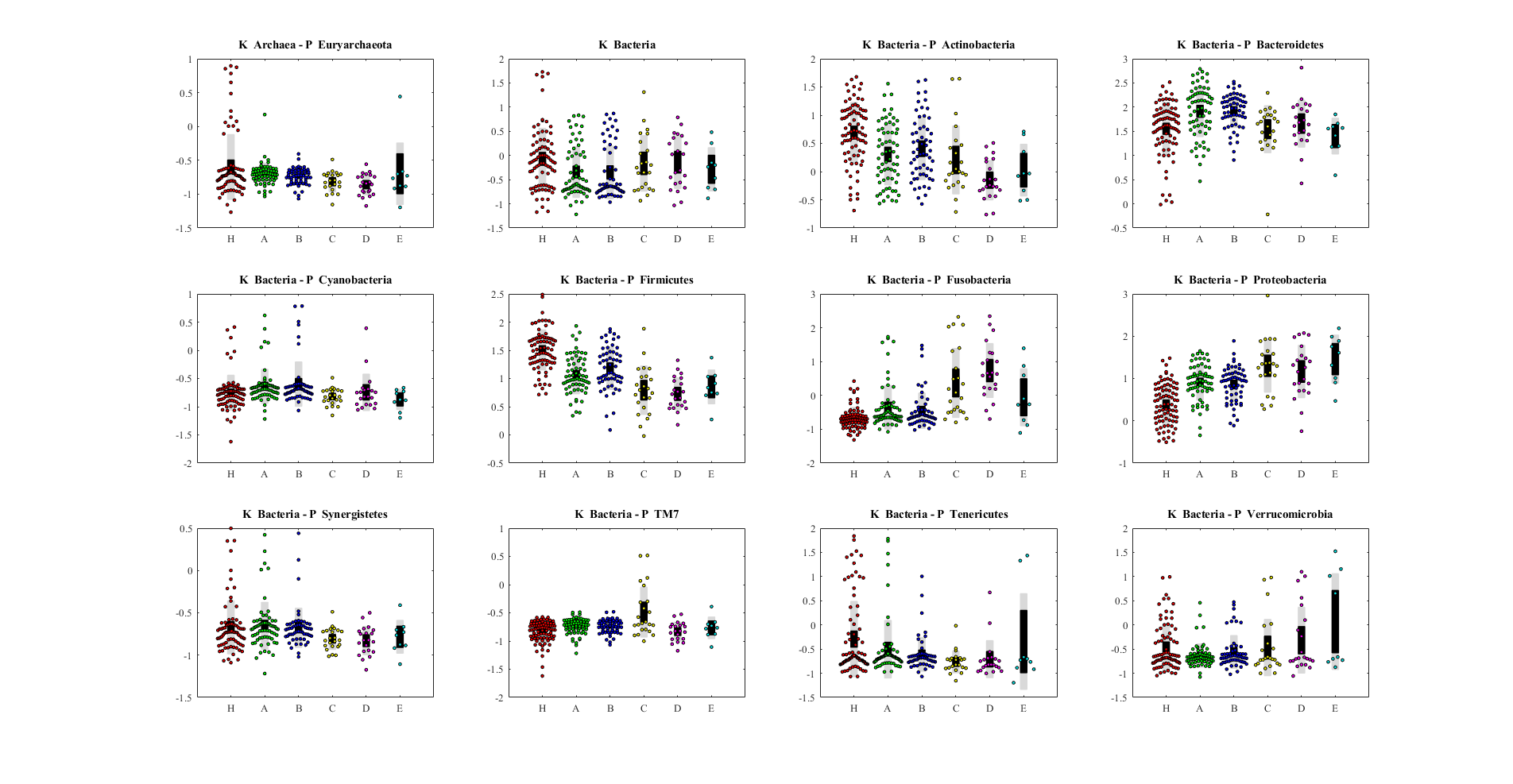

Supplement: FIG S2 [file msystems.01367-20-sf002.tif]

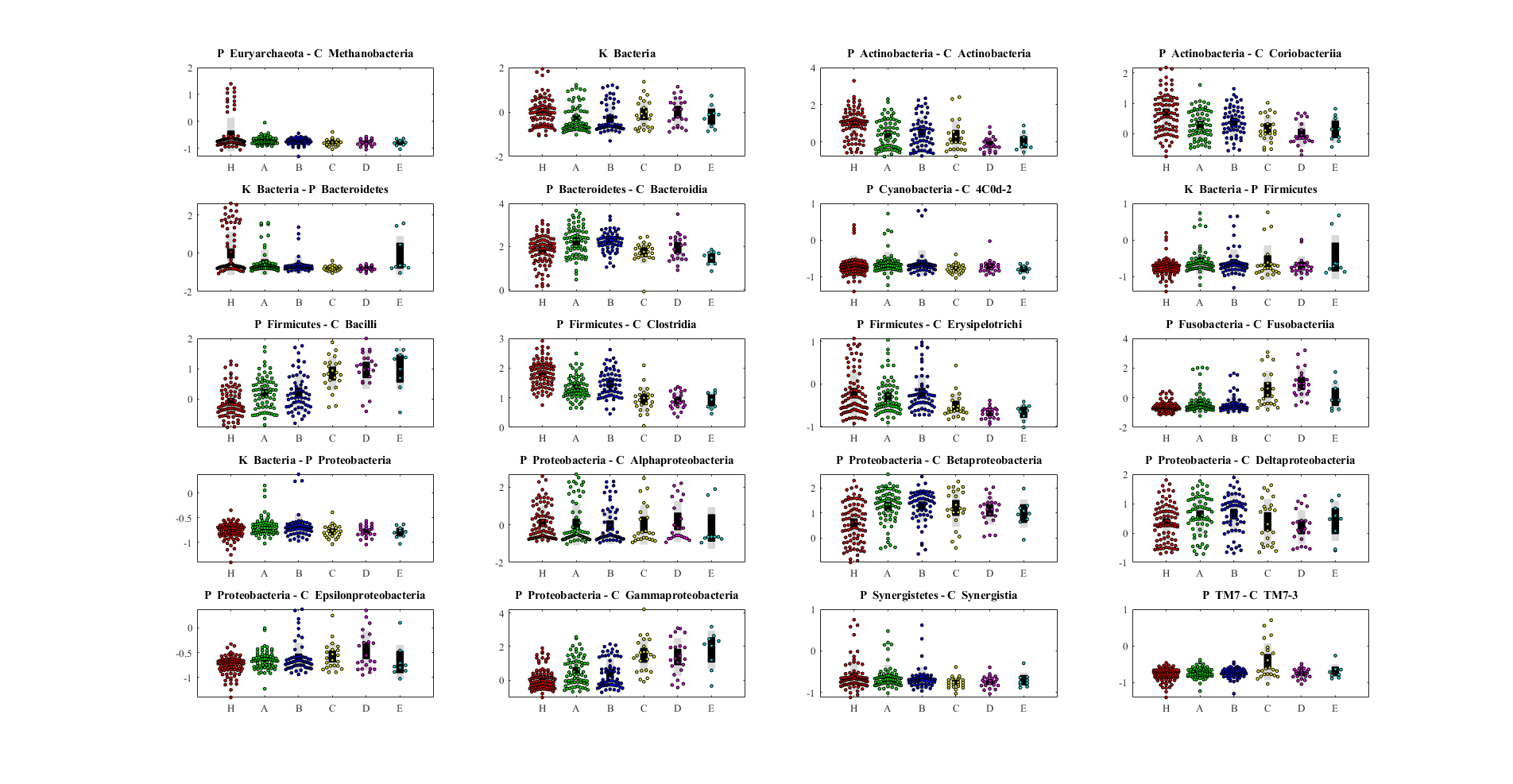

Supplement: FIG S3 [file msystems.01367-20-sf003.tif]

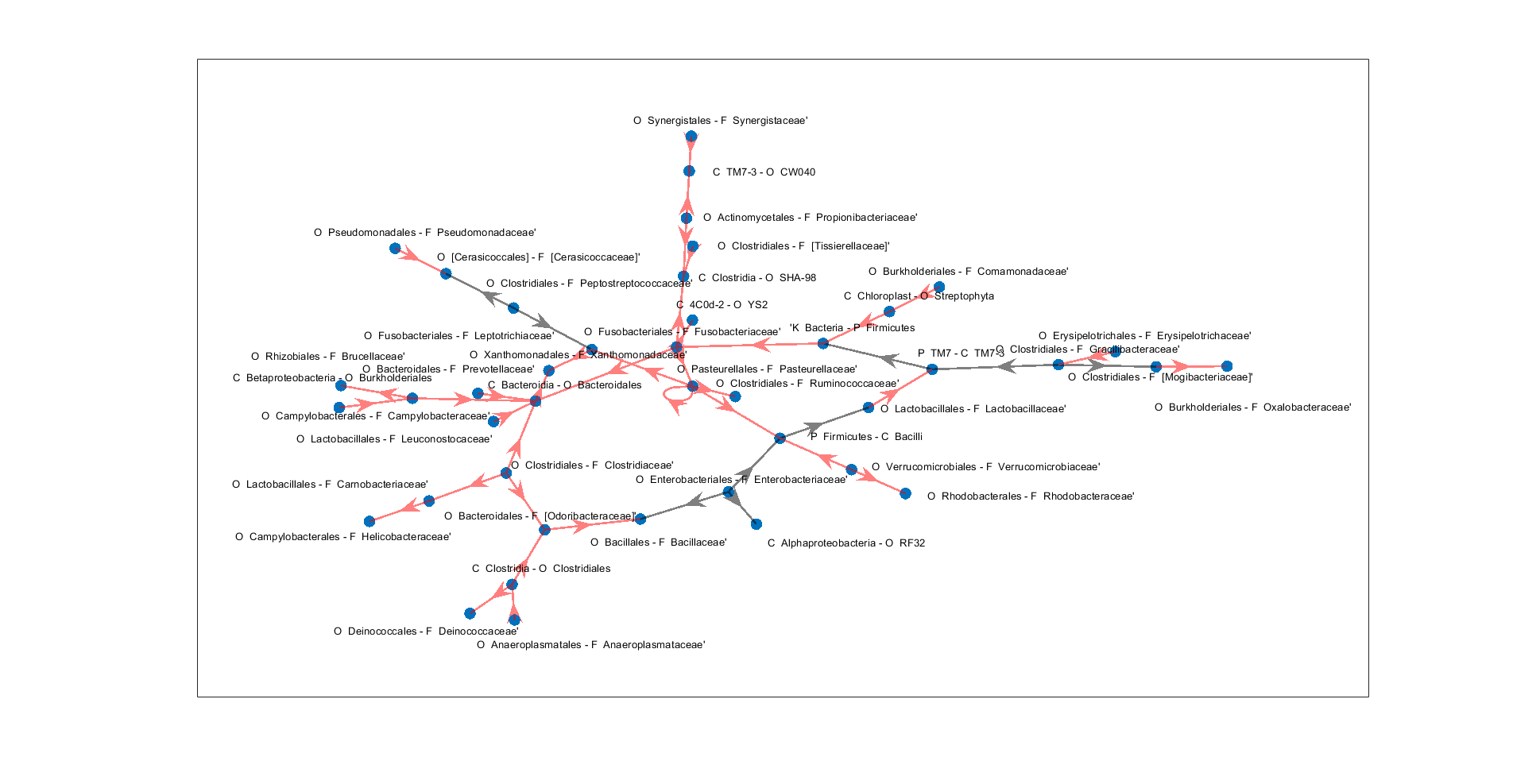

Supplement: FIG S4 [file msystems.01367-20-sf004.tif]
